# Downregulation of CSN6 attenuates papillary thyroid carcinoma progression by reducing Wnt/*β*‐catenin signaling and sensitizes cancer cells to FH535 therapy

**DOI:** 10.1002/cam4.1272

**Published:** 2018-01-17

**Authors:** Duo Wen, Tian Liao, Ben Ma, Ning Qu, Rong‐Liang Shi, Zhong‐Wu Lu, Yu‐Long Wang, Wen‐Jun Wei, Qing‐Hai Ji

**Affiliations:** ^1^ Department of Head and Neck Surgery Fudan University Shanghai Cancer Center Shanghai 200032 China; ^2^ Department of Oncology Shanghai Medical College Fudan University Shanghai 200032 China

**Keywords:** CSN6, EMT, FH535, papillary thyroid cancer, proliferation, Wnt/*β*‐catenin signaling pathway

## Abstract

The incidence of thyroid cancer has increased worldwide at a rate higher than that of any other cancer. CSN6 is overexpressed in many types of cancers, and such expression is linked to oncogenic activity. However, the detailed biological functions of CSN6 in papillary thyroid cancer (PTC) have not been well characterized. We investigated CSN6 expression in PTC specimens and cell lines. We used short‐hairpin RNA‐mediated gene silencing to explore the biological effects of CSN6 depletion in PTC cells. The combined effects of CSN6 silencing and FH535 therapy were assessed in terms of cell viability. The mechanism by which CSN6 regulated *β*‐catenin expression was also analyzed. CSN6 levels were determined by real‐time polymerase chain reaction (PCR) (mRNA), Western blotting, and immunochemistry (protein). The CCK‐8 and migration assays and orthotopic xenograft transplantation were used to investigate the biological effects of CSN6. We assessed the combined effects of CSN6 silencing and FH535 on cell viability in vitro. We also analyzed the relationship between the CSN6 level and clinical pathological status. CSN6 was overexpressed in human PTCs, and loss of CSN6 attenuated tumor proliferation and migration both in vitro and in vivo. CSN6 stabilized *β*‐catenin and facilitated the epidermal‐to‐mesenchymal transition (EMT) in PTC cells. CSN6 positively regulated *β*‐catenin expression in a *β*‐Trcp‐dependent manner and triggered expression of several EMT‐related genes regulated by *β*‐catenin. CSN6 silencing sensitized PTC cells to FH535 therapy via downregulation of the Wnt/*β*–catenin signaling pathway. Finally, in PTC patients, the level of CSN6 was significantly (inversely) correlated with tumor size, the presence of multifocal lesions, and TNM stage. CSN6 overexpression in PTC is a strong indicator of enhanced tumor aggressiveness. CSN6 promotes PTC progression by inducing the EMT. CSN6 knockdown sensitizes PTC cells to FH535 therapy via downregulation of the Wnt/*β*–catenin signaling pathway.

## Introduction

Papillary thyroid cancer (PTC) is the most common endocrine malignancy and accounts for 80% of all cases of differentiated thyroid cancer worldwide 1. As is true of most malignancies, thyroid carcinomas are usually associated with the triggering of aberrant cell proliferation by genetic alterations 2. However, no detailed picture of the pathways deregulated in PTC is yet available, and identification of molecular alterations would help guide treatment and improve clinical care.

The COP9 signalosome, generally termed the CSN, is a conserved multiprotein complex playing diverse roles in protein ubiquitination, transcriptional activation, signal transduction, and tumorigenesis 3. The CSN consists of eight subunits termed CSN1‐CSN8 4. CSN6, a subunit of the COP9 signalosome complex, is overexpressed in many types of cancer including glioblastoma, breast cancer, and myelomas; the CSN6 level is linked to oncogenic activity 5, 6, 7. However, the detailed biological roles played by CSN6 in PTC have not been well characterized.

*β*‐catenin, which is a subunit of the cadherin protein complex that induces tumorigenesis by promoting cell proliferation and blocking cell differentiation, acts as an intracellular signal transducer in the Wnt signaling pathway 8, 9. One important role for *β*‐catenin is induction of a morphogenic change in epithelial cells 10. During this process, epithelial cells cease to express E‐cadherin, Zonula occludens 1 (ZO1) protein, and cytokeratin 11, 12, 13 and begin to synthesize vimentin, alpha smooth muscle actin (ACTA2), and fibroblast‐specific protein 1 (FSP1) 14, 15, 16. *β*‐catenin expression is disturbed in many types of cancer. However, the means by which *β*‐catenin is overexpressed in PTC remains poorly characterized.

In this study, we show that CSN6 is overexpressed in PTC and suggest that CSN6 facilitates the epithelial–mesenchymal transition (EMT) of PTC cells via regulation of *β*‐catenin expression. Furthermore, we demonstrate that CSN6 enhances *β*‐catenin stability and that the CSN6‐associated protein *β*‐Trcp negatively regulates *β*‐catenin stability. Finally, we show that CSN6 knockdown sensitizes PTC cells to FH535 therapy via downregulation of the Wnt/*β*–catenin pathway. These results reveal a new means of posttranslational control of *β*‐catenin expression and explain the role played by CSN6 during PTC tumorigenesis. The data may improve therapies for PTC.

## Materials and Methods

### Tissue specimens

A total of 114 thyroid cancer samples were obtained from patients who underwent thyroid cancer surgery between 2012 and 2015 at Shanghai Cancer Center, Fudan University. Tissue specimens were frozen in liquid nitrogen immediately after surgical resection and stored at −80°C until use. Final histological classification was obtained from paraffin‐embedded sections. For the use of the clinical materials for research purposes, the Institutional Research Ethics Committee approved the study, and prior patient consent was required.

### Chemicals

FH535 was purchased from Selleck, and dissolved in DMSO at a concentration of 50 mmol/L as a stock solution and used by diluting in DMSO to give a final concentration of 50 *μ*mol/L 17.

### Cell lines and transfections

Three papillary thyroid carcinoma cell lines (TPC‐1, K1, BCPAP) and a normal human thyroid epithelial cell line (Nthy‐ori 3‐1) were used. Nthy‐ori‐3 and TPC‐1 cell lines were provided by Dr. Haixia Guan. K1 was gifted by Dr. Zebing Liu. BCPAP cell lines were purchased from the Institute of Biochemistry and Cell Biology at the Chinese Academy of Sciences (Shanghai, China). Nthy‐ori3‐1, BCPAP, TPC‐1, and K1 were grown in RPMI1640 media supplemented with 10% FBS (Invitrogen, Carlsbad, CA, USA) and 1% penicillin‐streptomycin (10,000 units’ penicillin and 10 mg streptomycin per mL in 0.9% NaCl, Sigma‐Aldrich) at 37°C with 5% CO_2_. Knockdown CSN6 in TPC‐1 and K1 was done using lentiviral infection: HEK‐293T cells were con‐transfected with pLKO.1‐CSN6 constructs and packaging plasmids, respectively. The virus‐containing medium released from HEK‐293T cells were utilized to infect the TPC and K1 cells to establish CSN6‐shRNA TPC cells and CSN6‐shRNA K1 cells, followed by 2 *μ*g/mL puromycin (Sigma‐Aldrich) selection.

### RNA extraction and RT‐PCR

Total RNA was extracted from cultured cells with TRIzol Reagent (Invitrogen, Inc.). 1 *μ*g of total RNA was used for first‐strand DNA synthesis employing a PrimeScriptTM RT reagent kit (Takara Bio, Inc., Japan). Real‐time PCR was performed in triplicate by according to the SYBR Green PCR method, with a SYBR Premix Ex TaqTM kit (Takara Bio, Inc., Japan) and in accordance with the manufacturer's instructions. The primers for the interested genes were synthesized by Sangon Company (Sangon Biotech Co., Ltd., Shanghai, China) (Table 1). The *β*‐actin was used as internal control for messenger RNA (mRNA) assays. The threshold cycle (*C*_*t*_) values were analyzed using the comparative *C*_*t*_ (−Δ*C*_*t*_) method. The level of targets was obtained by normalizing to the endogenous reference and relative to a control.

**Table 1:** qRT‐PCR primers of target genes

Target genes	Forward sequence (5′ to 3′)	Reverse sequence (5′ to 3′)
CSN6	TCATCGAGAG CCCCCTCTTT	CCAATGCGTTCCGCTTCCT
Myc	ATCACAGCCCTCACTCAC	ACAGATTCCACAAGGTGC
Jun	TTCTATGACGATGCCCTCAACGC	GCTCTGTTTCAGGATCTTGGGGT
Sox9	GCTCTACTCCACCTTCACC	CTCTGTCACCATTGCTCTT
Met	ACCTTTGATATAACTGTTTACTTG	GCTTTAGGGTGCCAGCATTTT
Mdm2	ATGAAAGCCTGGCTCTGTGT	GAAGCCAATTCTCACGAAGG
*β*‐catenin	CACGATGGCTACTCAAGCTG	CTGCGGATCCTTACAGTCAAAC
vimentin	GAGTCCACTGAGTACCGGAGAC	TGTAGGTGGCAA TCTCAATGTC
*β*‐actin	TGACGTGGACATCCGCAAAG	CTGGAAGGTGGACAGCGAGG

### Western blotting

Lysates were obtained from 1 × 10^6^ cultured cells with a mixture of ProteoJET Mammalian Cell Lysis Reagent (Fermentas, Inc.); phenylmethanesulfonyl fluoride (Roche, Inc.); and PhosSTOP (Roche, Inc.). About 20 *μ*g protein was extracted from each sample, separated by 10% sodium dodecyl sulfate–polyacrylamide gel electrophoresis (SDS‐PAGE). After being blocked in 5% nonfat milk, the interested protein was probed with antibody either against human CSN6 (1:1000; Cell Signaling Technology), *β*‐catenin (1:1000; abcam), ZO‐1 (1:1000; abcam), Vimentin (1:1000; Cell Signaling Technology), GAPDH (1:5000; Abcam), *β*‐Trcp; (1:1000; abcam), p38 (1:1000; abcam), PARP*γ* (1:1000; abcam), and Cyclin D1 (1:1000; abcam), then incubated with goat anti‐rabbit or anti‐mouse IgG (1:5000 for both; Jackson ImmunoResearch Laboratories); and detected with enhanced chemiluminescence reagents (Thermo Fisher Scientific). The bands were visualized using 1‐stepTM NBT/BCIP reagents (Thermo Fisher Scientific, Rockford, IL, USA) and detected by the Alpha Imager (Alpha Innotech, San Leandro, CA, USA).

### Cellular viability assays

Cell proliferation was determined using a cell counting kit‐8 (CCK‐8) assay according to the manufacturer's protocol. Briefly, cells were seeded into 96‐well plates at 6 × 10^3^ cells/well. An aliquot of 10 *μ*L CCK‐8 solution was added to each well, and the plate was incubated for 4 h at 37°C. At the indicated time points, the absorbance at 450 nm was measured using a spectrophotometer. For each group, data from five wells were pooled.

### Immunohistochemical staining

IHC was carried out according to the manufacturer's protocol. Briefly, formalin‐fixed and paraffin‐embedded tissue sections were deparaffinized in xylene and hydrated through descending concentrations of ethanol before being placed in blocking solution to inhibit endogenous peroxidase activity. The slides were incubated with primary antibodies at 4°C overnight. A horseradish peroxidase‐conjugated rabbit or mouse secondary antibody was added for 60 min at room temperature, followed by 3, 3′‐diaminobenzidine (DAB) development (DAB Substrate Chromogen System, Dako) and hematoxylin and eosin (H&E) as per standard staining protocol. Slides were fixed and images obtained with the Olympus IX71 inverted microscope using the DP2‐BSW Olympus image acquisition software system. The results were confirmed by two experienced pathologists who were blinded to the clinicopathologic data of the patients. The staining results were calculated on the basis of the percentage of tumor cell nuclei stained (0, no staining; 1, ≦10%; 2, 10–50%, and 3, >50%) and the staining intensity (0, negative; 1, weak; 2, moderate; and 3, strong). An overall score of 1–5 designated low expression, and an overall score of 6–9 designated high expression for both CSN6‐ and EMT‐related genes in PTC tissues.

### Immunofluorescent staining and confocal laser scanning microscopy

Cells were cultured as described above. Then cells were washed twice with cold PBS and fixed in 4% paraformaldehyde for 20 min at 4°C. After three PBS washes, cells were permeabilized with 0. 1% Triton X–100 for 5 min and incubated in PBS containing 1% BSA for 30 min, followed by overnight incubation at 4°C with primary antibodies. After successive washes, Alexa Fluor 594‐conjugated secondary antibody (1:500; Invitrogen) was added and incubated for 1 h at room temperature. Then the cells were washed with PBS and nuclei were labeled with DAPI for 2 min. Samples were mounted with immunofluorescence mounting medium (DakoCytomation, Carpinteria, CA) and analyzed by confocal microscopy. Confocal laser‐scanning microscopy was performed using a Leica TCS SP2 laser‐scanning spectral confocal microscope and Carl Zeiss LSM 780 NLO. Excitation was with an argon laser, emitting 488 nm; a krypton laser, emitting at 568 nm; and a helium/neon laser, emitting at 633 nm. Data were acquired and analyzed with Leica confocal software. All two‐ or three‐color images were acquired using a sequential scan mode.

### Animal experiments

Five‐week‐old BALB/c nude mice were obtained from the Shanghai experimental animal center (Shanghai, China). Briefly, 3 × 10^6^ cells were subcutaneously injected into the right back skin area of BALB/c nude mice. The size of tumors was measured by Vernier caliper twice a week. After 28 days, mice were killed, and tumor tissues were collected, photographed, and examined. Paraffin‐embedded tissues were sectioned for IHC analysis. Part of tumor tissue was frozen in liquid nitrogen for following experiments. Animal experiments have been preapproved by the Shanghai Cancer Center, Fudan University and all procedures were performed in accordance with the National Institutes of Health Guide for the Care and Use of Laboratory Animals.

### Statistics

Statistical analyses were performed with SPSS version 18.0 for Windows. For comparison among the groups, a Student's *t* test, Chi‐Square test or a Fisher's Exact test was performed, and *P *<* *0.05 was defined as statistically significant. The data and error bars report the means ± SEM. Each experiment was repeated at least three times.

## Results

### CSN6 is overexpressed in human PTC

**Figure 1:**
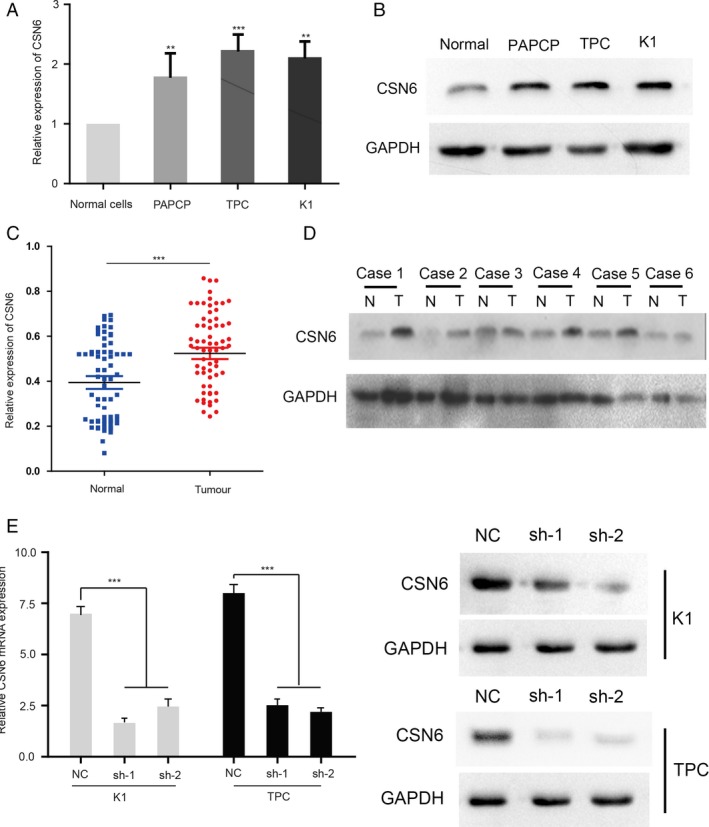
(A) Relative expression of CSN6 in 60 PTC patients; (B) CSN6 expression in 6 pairs of PTC tissues (T) and adjacent non‐PTC tissues (N); (C) Relative expression of CSN6 in Nthy‐ori3‐1, BCPAP, TPC‐1, and K1 cells; (D) The protein expression of CSN6 in Nthy‐ori3‐1, BCPAP, TPC‐1, and K1 cells. (D and E) Real‐time PCR and Western blot analysis of CSN6 mRNA and protein level in control (NC), and knockdown (sh‐1, sh‐2) PTC cells. **P < 0.01, ***P < 0.001.

Quantitative real‐time PCR analysis of CSN6 expression levels in 60 paired samples of PTC tissue and adjacent normal tissue revealed that CSN6 was expressed to a significantly greater extent in cancerous than normal tissue (Fig. 1A). Western blotting data on CSN6 expression levels in PTC tissue were consistent with the results of real‐time polymerase chain reaction (PCR) (Fig. 1B). Immunohistochemical tissue microarray data revealed that CSN6 expression in PTC was higher than that in normal tissue in 66 of the 80 (82.5%) paired samples. Furthermore, Western blotting analysis of endogenous CSN6 expression in one thyroid cell line (Nthy‐ori3‐1) and three PTC cell lines revealed that CSN6 was overexpressed in the PTC cell lines but not in normal thyroid cells (Fig. 1C and D). Therefore, CSN6 was overexpressed in human PTC.

### Loss of CSN6 attenuates tumor proliferation and migration

**Figure 2:**
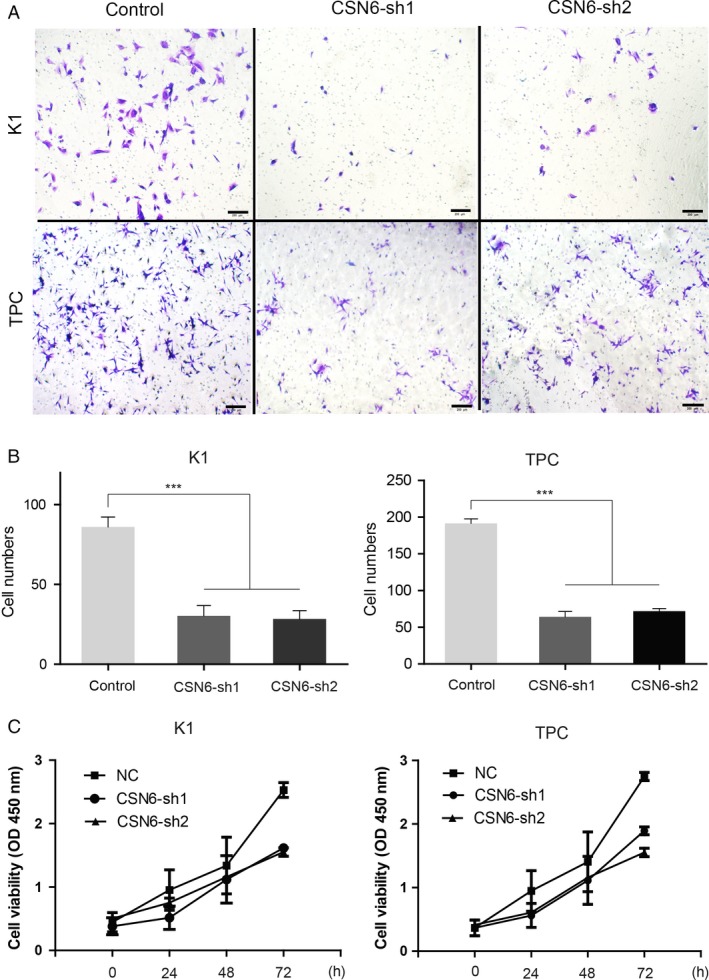
(A and B) Migration assays of K1 and TPC‐1 cells with indicated treatment; (C) Cell viability of K1 and TPC‐1 were examined by CCK‐8 assay. Data are mean ± SEM and are representative of three independent experiments. ***P < 0.001.

Tumor proliferation, migration, and invasion are the most important steps in the cascade of tumor metastasis. Therefore, we first investigated the effect of CSN6 on PTC proliferation. Two human PTC cell lines, TPC‐1 and K1, were subjected to stable transfection with sh‐CSN6 and the sh‐CSN6 vector (control). As shown in Figure 1E, both the CSN6 mRNA and protein levels were reduced in TPC‐1 and K1 cells following transfection with the CSN6 shRNA plasmid (Fig. 1E). The roles played by CSN6 in PTC proliferation and migration were next examined. The migration capacities of K1 and TPC‐1 cells were reduced by more than 2.3‐ and 3.6‐fold, respectively, by CSN6 shRNA, compared with those of the control cells (Fig. 2A and B). These results suggest that CSN6 robustly modulates PTC migration and invasiveness. We then used the CCK‐8 method to measure cell proliferation. The results suggested that, compared to the control, the silencing of CSN6 expression inhibited the proliferation of K1 and TPC‐1 cells (Fig. 2C).

**Figure 3:**
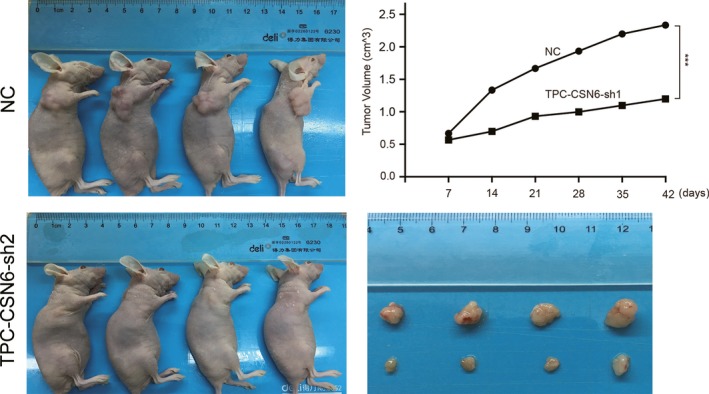
Images showing the primary tumor volume in the recipient mice. ***P < 0.001.

Next, we investigated the effects of CSN6 on PTC proliferation in vivo via orthotopic xenograft transplantation of TPC‐1 cell lines. We found that, at day 28 postinjection, the mean tumor volume in CSN6‐sh2‐implanted animals was threefold that in NC‐implanted animals (Fig. 3). Thus, loss of CSN6 expression inhibits PTC proliferation and migration.

### CSN6 positively regulates *β*‐catenin protein stability and facilities the EMT in PTC cells

**Figure 4:**
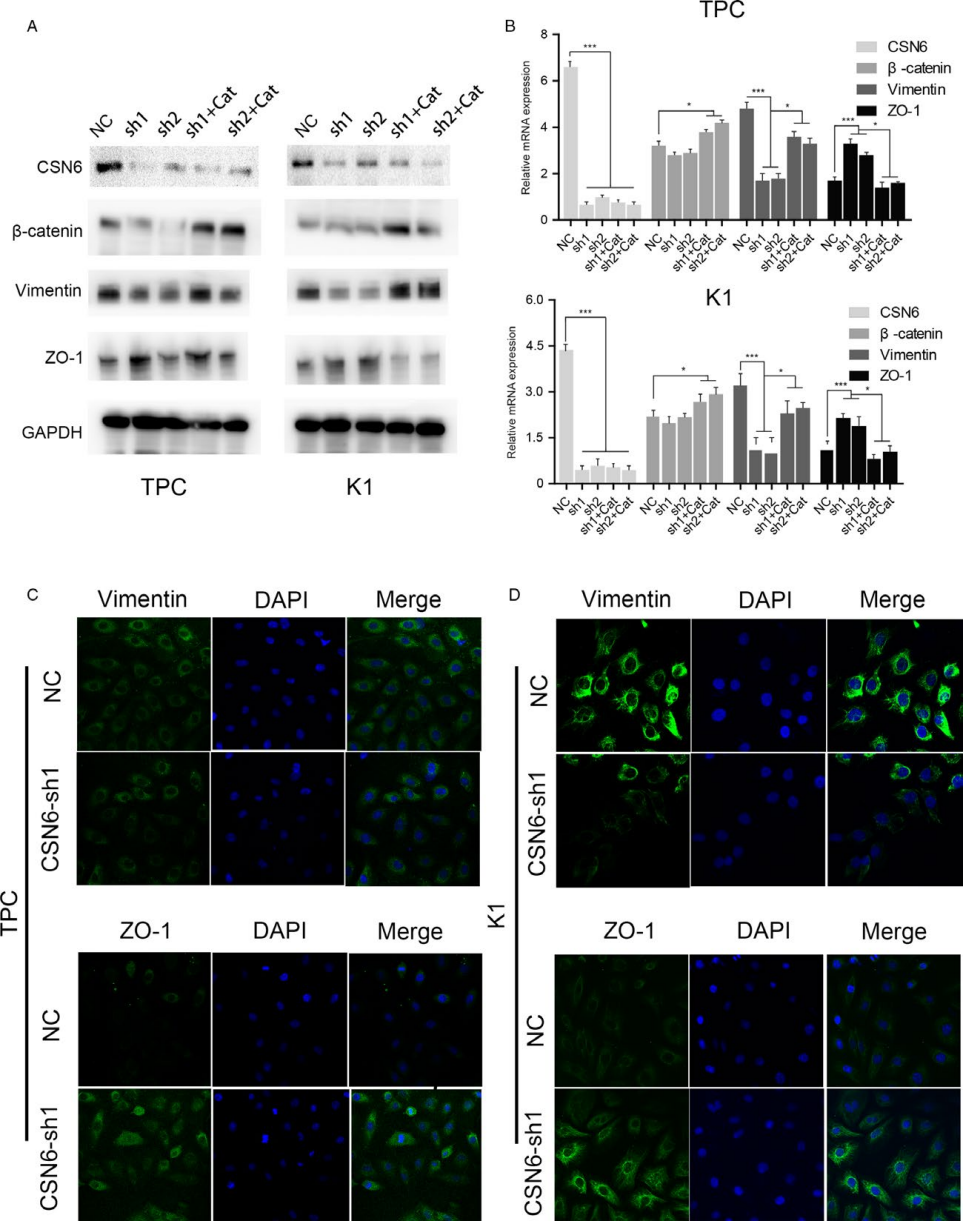
(A) Western bolt analysis of proteins in treated PTC cells; (B) The relative mRNA expression in treated PTC cells; (C and D) the internalization intracellular distribution of vimentin and ZO‐1 was gained by confocal laser scanning microscopy observations. *P < 0.05, ***P < 0.001.

To identify possible mediators of the effects of CSN6, we first measured the levels of mRNAs encoding EMT‐related transcription factors. CSN6 downregulation in K1 and TPC‐1 cells significantly increased ZO‐1 gene expression and decreased that of the vimentin gene (Fig. 4A and B). CSN6 and *β*‐catenin proteins levels were positively correlated in the PTC cell lines (Fig. 4A). Furthermore, continuous knockdown of CSN6 expression in K1 and PTC cells decreased the *β*‐catenin protein levels, whereas the restoration of *β*‐catenin expression using an expression vector (Cat‐pcDNA, Cat) increased expression of both vimentin mRNA and protein to some extent (Fig. 4A).

Further insight into the intracellular distributions of vimentin and ZO‐1 was gained by confocal laser scanning microscopy. The green and blue colors indicate vimentin or ZO‐1 and nuclei (stained with Hoechst 33342), respectively. The immunofluorescence assay data show that vimentin expression obviously declined in both TPC and K1 cells treated with CSN6‐sh1 compared with in cells transfected with the blank plasmid (Fig. 4C and D). In contrast, ZO‐1 expression increased in CSN6‐sh1‐treated cells compared with NC (Fig. 4C and D). Together, the results suggest that CSN6 may positively regulate *β*‐catenin transcription, thereby mediating the EMT.

### CSN6 positively regulates *β*‐catenin expression in a *β*‐Trcp‐dependent manner and triggers the expression of several EMT‐related genes regulated by *β*‐catenin

It has been reported that *β*‐Trcp regulates *β*‐catenin stability, and CSN6 is known to regulate *β*‐catenin stability 18, 19. Thus, we explored whether CSN6 negatively regulated *β*‐Trcp expression to stabilize *β*‐catenin in PTC cells. As expected, we found that the *β*‐catenin level decreased and that of *β*‐Trcp increased when cells were treated with CSN6‐shRNA (CSN6 knockdown) (Fig 6A and B). Furthermore, we found that the level of mRNA encoding *β*‐catenin was not affected by CSN6 knockdown (Fig. 4B). Western blotting revealed that the CSN6‐mediated *β*‐Trcp downregulation in K1 cells could be rescued by the proteasome inhibitor MG132 (Fig. 6C). These results suggest that CSN6 regulates *β*‐Trcp and *β*‐catenin expression at the posttranscriptional level in PTC cells. Furthermore, we found that continual silencing of *β*‐Trcp expression in CSN6‐knockdown cells partly rescued the expression of several genes regulated by *β*‐catenin (Fig. 6D and E). Taken together, the results show that CSN6 overexpression stabilized *β*‐catenin in a *β*‐Trcp‐dependent manner, subsequently increasing the expression of *β*‐catenin‐controlled genes.

**Figure 5:**
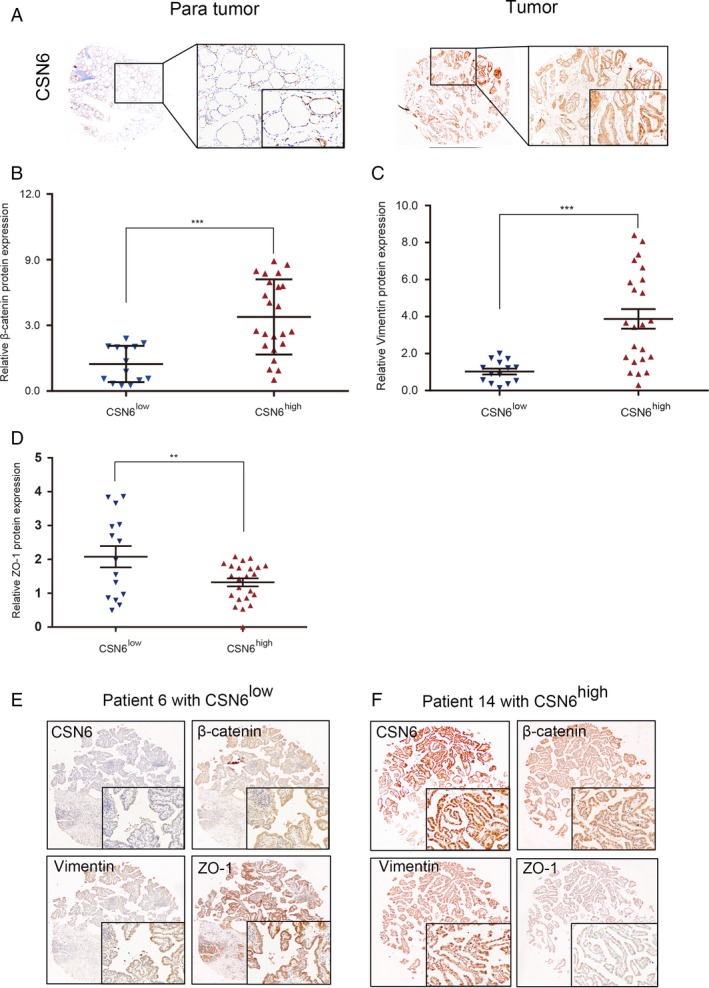
(A) Representative immunostaining images in PTC tissue arrays using CSN6‐specific antibodies; (B, C and D) Relative levels of β‐catenin, vimentin and ZO‐1 in patients with low and high CSN6 expression levels; (E and F) Images to visualize positive staining of β‐catenin, vimentin and ZO‐1 in patients with low and high CSN6 expression levels. The slides are constructed by serial section method. Data are mean ± SEM and are representative of three independent experiments. **p<0.01, ***p<0.001.

**Figure 6:**
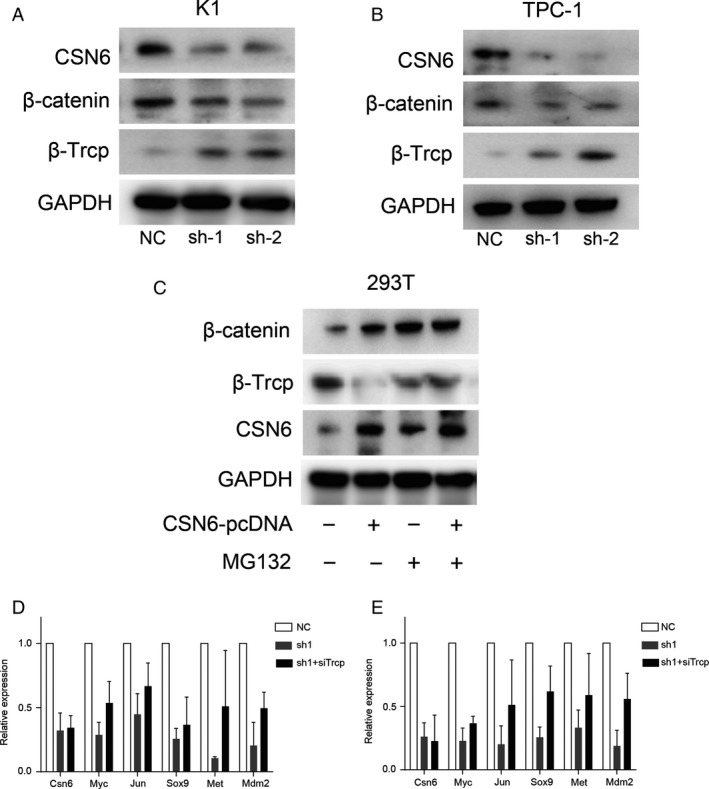
(A and B) Western bolt analysis of proteins in CSN6 knockdown PTC cells and control (NC); (C) Western bolt analysis of proteins in treated cells and their corresponding control group. (D and E) the expression of several EMT‐related genes regulated by β‐catenin.

### Knockdown of CSN6 sensitizes PTC cells to FH535 therapy via downregulation of the Wnt/*β*–catenin signaling pathway

**Figure 7:**
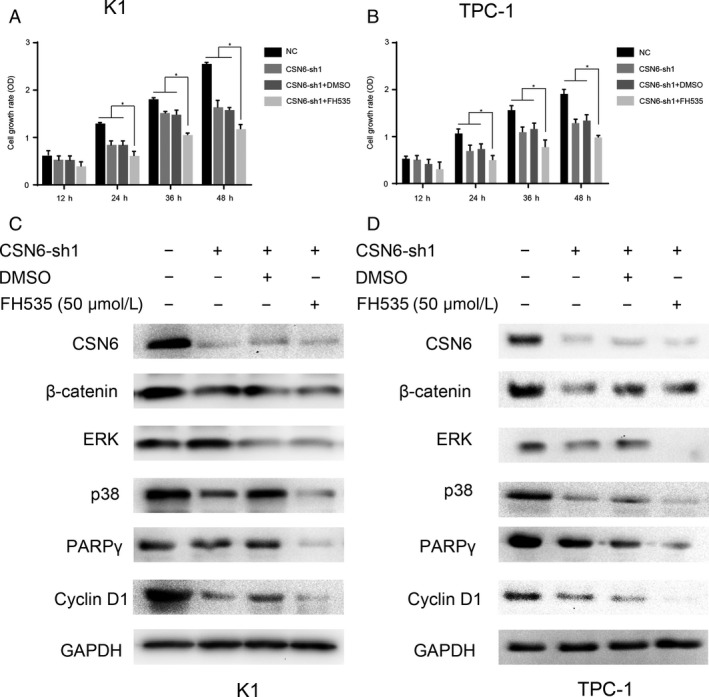
(A and B) Cell viability of K1 and TPC‐1 were examined by CCK‐8 assay. (C and D) Western bolt analysis of proteins in CSN6 knockdown, DMSO and FH535 treated cells. *p<0.05.

Abnormal activation of the Wnt/*β*–catenin pathway has been observed in various cancers, a significant number of which exhibit overaccumulation of *β*‐catenin protein in cancer cells 8, 20. Therefore, we explored whether CSN6 silencing in PTC cells would sensitize such cells to Wnt/*β*‐catenin inhibitor therapy. To this end, we used FH535, which exhibits a selective anti‐proliferation effect on certain cancer cells exhibiting a high level of expression of the Wnt/*β*–catenin pathway 17. We examined the effects of FH535 on proliferation of CSN6‐shRNA‐treated K1 and TPC‐1 cells. Cells were grown in medium 1640 and were or were not treated with 50 *μ*mol/L FH535. As shown in Figure 7A and B, FH535 more strongly inhibited the proliferation of CSN6‐silenced PTC cell lines than control and untreated CSN6‐silenced cells. Next, we examined the effects of FH535 on Wnt*β*‐catenin signaling in PTC cells. We measured the levels of several markers that are known to be regulated by Wnt/*β*‐catenin signaling; these include vimentin, p38, PAPR*γ*, and cyclin D1 21, 22, 23. The levels of these proteins were lower in the CSN6‐sh2 group than in the control group as shown in Figure 7C and D. Moreover, the protein expression levels in the FH535‐treated group were lower than those in the nontreated group. These results show that CSN6 knockdown sensitizes PTC cells to FH535 therapy via downregulation of the Wnt/*β*–catenin pathway. Thus, FH535 is potentially of therapeutic value for treatment of PTC associated with low‐level CSN6 expression and, thus, an overactive Wnt/*β*–catenin pathway.

### CSN6, *β*‐catenin, ZO‐1, and vimentin expression levels in PTC patients

Based on the similar results of experiments using PTC cell lines and the xenograft model, it appeared that reduced CSN6 expression impaired *β*‐catenin stability in a *β*‐Trcp‐dependent manner, thus promoting the EMT and cancer cell proliferation. To explore whether this was true in PTC patients, we first used real‐time PCR to seek relationships between the CSN6 level and the clinical pathological status in 114 archived PTCs. The clinicopathological features of the patients are listed in Table 2. Tumors from 22 patients who had lymph node metastases tended to express higher levels of CSN6 than did those from non‐metastatic patients. Additionally, the CSN6 level was significantly (inversely) correlated with tumor size and the presence of multifocal lesions. Thus, a significant association was evident between high‐level CSN6 expression and PTC dissemination.

**Table 2:** The relationship between CSN6 expression and clinicopathologic parameters in PTC patients

Clinicopathologic parameters	CSN6 expression		*P*‐value
	Low (%)	High (%)	
Age (years)			0.783
<45	20 (50.0)	35 (47.3)	
≥45	20 (50.0)	39 (52.7)	
Gender			0.627
Male	17 (42.5)	28 (37.8)	
Female	23 (57.5)	46 (62.2)	
Tumor size (cm)			0.037^a^
≤1	14 (35.0)	41 (55.4)	
>1	26 (65.0)	33 (44.6)	
Location of the primary tumors			
Solitary lesion			0.232
Upper third	6 (15.0)	17 (23.0)	
Middle third	22 (55.0)	26 (35.1)	
Lower third	8 (20.0)	22 (29.7)	
Isthmus	4 (10.0)	9 (12.2)	
Multifocal lesions			0.026^a^
Positive	8 (20.0)	30 (40.5)	
Negative	32 (40.0)	44 (59.5)	
Bilateral			0.479
Positive	5 (12.5)	13 (17.6)	
Negative	35 (87.5)	61 (82.4)	
Extrathyroidal extension			0.178
Positive	12 (30.0)	14 (18.9)	
Negative	28 (70.0)	60 (81.1)	
Lymph node metastasis			0.039^a^
Positive	5 (12.5)	22 (29.7)	
Negative	35 (87.5)	52 (70.3)	
TNM stage			0.126
I + II	28 (70.0)	61 (82.4)	
III + IV	12 (30.0)	13 (17.6)	

a *P* < 0.05. Chi‐squared test *P*‐value.

Next, we investigated whether the CSN6 level was associated with the protein levels of *β*‐catenin and other EMT biomarkers in PTC patients. Immunohistochemical assays revealed that the expression patterns of *β*‐catenin, ZO‐1, and vimentin differed between the 14 CSN6^low^ and 23 CSN6^high^ PTC patients. Higher levels of *β*‐catenin and vimentin were evident in the CSN6^high^ patients, whereas the ZO‐1 level was higher in the CSN6^low^ patients (Fig. 5B–D). Furthermore, tumors with low‐level CSN6 (e.g., the tumor of patient 6; CSN6^low^) tended to express low levels of *β*‐catenin and vimentin and a high level of ZO‐1, whereas tumors with high levels of CSN6 tended to express high levels of *β*‐catenin and vimentin and a low level of ZO‐1 (e.g., patient 14; CSN6^high^) (Fig. 5E and F).

## Discussion

In this study, we found that high expression of CSN6 in PTC is significantly correlated with tumor size, the presence of multifocal lesions, and TNM stage, suggested that CSN6 may play important role on the oncogenesis of PTC. In vitro and in vivo data showed that loss of CSN6 attenuated cell proliferation, migration, and invasion of PTC cells, confirming the vital function of CSN6 in PTC. Further mechanism study demonstrated that the role of CSN6 in PTC may result from the regulation of EMT via Wnt/*β*–catenin signaling pathway in a *β*‐Trcp‐dependent manner. This preclinical study is the first time to illustrate the involvement of CSN6 in PTC and explain the underlying mechanism of the role of CSN6 on PTC.

It is known that CSN6 has emerged as an important protein involved in cell cycle regulation, but its role in PTC remains unclear 24. We used an extensive collection of PTC tumors to show that CSN6 was highly expressed in a substantial proportion of such tumors, as shown both by evaluation of mRNA expression and tissue microarray studies. CSN6 expression was clearly associated with tumor size and lymph node metastasis status. CSN6 overexpression was positively correlated with *β*‐catenin protein expression in human PTC samples. These observations are consistent with those described in previous reports on other malignancies, such as colon and breast cancers 7, 18. In our present study, several lines of evidence suggest a close association between CSN6 expression and the malignant biological manifestations of PTC. First, the CSN6 protein and mRNA expression levels were much higher in PTC cells compared with normal thyroid epithelial cells. Second, functional assays showed that CSN6 inhibition significantly reduced both the motility and the proliferation of PTC cells. Third, using an orthopopic xenograft model, we found that CSN6 knockdown in implanted PTC cells markedly reduced the tumor size in nude mice. Thus, these findings strongly suggest that CSN6 plays a crucial role in regulating PTC progression and may serve as both a marker of PTC tumor aggressiveness and a predictor of prognosis in PTC patients.

The mechanisms by which CSN6 enhances various aspects of tumor malignancy remain poorly defined. One novel finding of this study is that CSN6 expression is closely associated with that of *β*‐catenin, which is known to be a key regulator of the EMT of various cancers 25, 26. During the EMT, the cell shape changes from cobblestone‐like (associated with tight cell–cell contact) into spindle‐shaped fibroblast‐like, with the loss of cell–cell contact and cell polarity 27. In tumor cells, this process can increase the proportion of mesenchymal‐like cells and decrease that of epithelial‐like cells, thus enhancing the invasiveness and metastatic potential of the tumor. *β*‐catenin plays an important regulatory role in the development of the EMT 8. CSN6 positively regulates *β*‐catenin expression. Thus, the positive correlation between CSN6 expression and the EMT may explain the role played by CSN6 in PTC malignancy 18. We found that when CSN6 was downregulated, the levels of *β*‐catenin and the mesenchymal marker vimentin decreased, whereas that of the epithelial marker ZO‐1 increased. These results suggest that CSN6 is indeed involved in the EMT in PTC cell lines. Next, we found that CSN6 regulated *β*‐catenin expression in a *β*‐Trcp‐dependent manner. Activating mutations in the Wnt/*β*–catenin pathway are well‐known carcinogenic mechanisms and may predict the response to treatment of various tumors 8. *β*‐catenin plays a key role in the Wnt/*β*–catenin signaling pathway. CSN6 stabilizes *β*‐catenin expression, thus triggering abnormal signaling 18. Human PTCs respond poorly to chemotherapeutic drugs 28, but those exhibiting CSN6 overexpression may respond well to Wnt/*β*‐catenin signaling inhibitors. In this study, we showed that the *β*‐catenin inhibitor FH535 significantly reduced proliferation of PTC cell lines. More importantly, CSN6 knockdown sensitized PTC cells to FH535 therapy via downregulation of the Wnt/*β*–catenin pathway. Thus, inhibition of the Wnt/*β*–catenin pathway may constitute targeted therapy for PTC.

Although we reported the function of CSN6 on proliferation, migration, and invasion of PTC for the first time, its effect on the progression of PTC need more evidence to prove, which is our further work. On the other hand, it needs more tumor samples to analyze its correlation with clinical parameters to confirm its vital role on the oncogenesis of PTC. The other limitation of our study is the molecular mechanism through which the CSN6 display on PTC.

In conclusion, we found that CSN6 overexpression in PTC was a strong indicator of enhanced tumor aggressiveness. CSN6 promotes PTC progression by inducing the EMT. CSN6 knockdown sensitizes PTC cells to FH535 therapy via downregulation of the Wnt/*β*–catenin signaling pathway. The evidence suggests that CSN6 may be a valid biomarker of PTC and a useful therapeutic target.

## Conflicts of Interest

No conflicts of interest to disclose.

## References

[1] Lubitz, C. C. , and J. A. Sosa . 2016 The changing landscape of papillary thyroid cancer: epidemiology, management, and the implications for patients. Cancer 122:3754–3759. 27517675 10.1002/cncr.30201

[2] REBAЇ, M. , and A. REBAЇ . 2016 Molecular genetics of thyroid cancer. Genet. Res. (Camb) 98:e7. 27174043 10.1017/S0016672316000057 PMC6865173

[3] Wei, N. , and X. W. Deng . 2003 The COP9 signalosome. Annu. Rev. Cell Dev. Biol. 19:261–286. 14570571 10.1146/annurev.cellbio.19.111301.112449

[4] Wolf, D. A. , C. Zhou , and S. Wee . 2003 The COP9 signalosome: an assembly and maintenance platform for cullin ubiquitin ligases? Nat. Cell Biol. 5:1029–1033. 14647295 10.1038/ncb1203-1029

[5] Choi, H. H. , C. Gully , C. H. Su , G. Velazquez‐Torres , P. C. Chou , C. Tseng , et al. 2011 COP9 signalosome subunit 6 stabilizes COP1, which functions as an E3 ubiquitin ligase for 14‐3‐3sigma. Oncogene 30:4791–4801. 21625211 10.1038/onc.2011.192 PMC3358116

[6] Hou, J. , Q. Deng , J. Zhou , J. Zou , Y. Zhang , P. Tan , et al. 2016 CSN6 controls the proliferation and metastasis of glioblastoma by CHIP‐mediated degradation of EGFR. Oncogene 36:1134–1144. 27546621 10.1038/onc.2016.280

[7] Zhao, R. , S. C. Yeung , J. Chen , T. Iwakuma , C. H. Su , B. Chen , et al. 2011 Subunit 6 of the COP9 signalosome promotes tumorigenesis in mice through stabilization of MDM2 and is upregulated in human cancers. J. Clin. Invest. 121:851–865. 21317535 10.1172/JCI44111 PMC3049400

[8] Clevers, H. 2006 Wnt/beta‐catenin signaling in development and disease. Cell 127:469–480. 17081971 10.1016/j.cell.2006.10.018

[9] Freese, J. L. , D. Pino , and S. J. Pleasure . 2010 Wnt signaling in development and disease. Neurobiol. Dis. 38:148–153. 19765659 10.1016/j.nbd.2009.09.003 PMC2854277

[10] Gumbiner, B. M. 1996 Cell adhesion: the molecular basis of tissue architecture and morphogenesis. Cell 84:345–357. 8608588 10.1016/s0092-8674(00)81279-9

[11] Chilosi, M. , A. Calio , A. Rossi , E. Gilioli , F. Pedica , L. Montagna , et al. 2016 Epithelial to mesenchymal transition‐related proteins ZEB1, beta‐catenin, and beta‐tubulin‐III in idiopathic pulmonary fibrosis. Mod. Pathol. 30:26–38. 27586205 10.1038/modpathol.2016.147

[12] Itoh, M. , A. Nagafuchi , S. Moroi , and S. Tsukita . 1997 Involvement of ZO‐1 in cadherin‐based cell adhesion through its direct binding to alpha catenin and actin filaments. J. Cell Biol. 138:181–192. 9214391 10.1083/jcb.138.1.181 PMC2139940

[13] Nelson, W. J. , and R. Nusse . 2004 Convergence of Wnt, beta‐catenin, and cadherin pathways. Science 303:1483–1487. 15001769 10.1126/science.1094291 PMC3372896

[14] Lee, H. W. , Y. M. Park , S. J. Lee , H. J. Cho , D. H. Kim , J. I. Lee , et al. 2013 Alpha‐smooth muscle actin (ACTA2) is required for metastatic potential of human lung adenocarcinoma. Clin. Cancer Res. 19:5879–5889. 23995859 10.1158/1078-0432.CCR-13-1181

[15] Mendez, M. G. , S. Kojima , and R. D. Goldman . 2010 Vimentin induces changes in cell shape, motility, and adhesion during the epithelial to mesenchymal transition. FASEB J. 24:1838–1851. 20097873 10.1096/fj.09-151639 PMC2874471

[16] Okada, H. , T. M. Danoff , R. Kalluri , and E. G. Neilson . 1997 Early role of Fsp1 in epithelial‐mesenchymal transformation. Am. J. Physiol. 273:F563–F574. 9362334 10.1152/ajprenal.1997.273.4.F563

[17] Iida, J. , J. Dorchak , J. R. Lehman , R. Clancy , C. Luo , Y. Chen , et al. 2012 FH535 inhibited migration and growth of breast cancer cells. PLoS ONE 7:e44418. 22984505 10.1371/journal.pone.0044418 PMC3439405

[18] Fang, L. , W. Lu , H. H. Choi , S. C. Yeung , J. Y. Tung , C. D. Hsiao , et al. 2015 ERK2‐dependent phosphorylation of CSN6 is critical in colorectal cancer development. Cancer Cell 28:183–197. 26267535 10.1016/j.ccell.2015.07.004 PMC4560098

[19] Hart, M. , J. P. Concordet , I. Lassot , I. Albert , R. Del los Santos , H. Durand , et al. 1999 The F‐box protein beta‐TrCP associates with phosphorylated beta‐catenin and regulates its activity in the cell. Curr. Biol. 9:207–210. 10074433 10.1016/s0960-9822(99)80091-8

[20] Fodde, R. , and T. Brabletz . 2007 Wnt/beta‐catenin signaling in cancer stemness and malignant behavior. Curr. Opin. Cell Biol. 19:150–158. 17306971 10.1016/j.ceb.2007.02.007

[21] Philipp, I. , R. Aufschnaiter , S. Ozbek , S. Pontasch , M. Jenewein , H. Watanabe , et al. 2009 Wnt/beta‐catenin and noncanonical Wnt signaling interact in tissue evagination in the simple eumetazoan Hydra. Proc. Natl Acad. Sci. USA 106:4290–4295. 19237582 10.1073/pnas.0812847106 PMC2646623

[22] Tennis, M. A. , M. M. Van Scoyk , S. V. Freeman , K. M. Vandervest , R. A. Nemenoff , and R. A. Winn . 2010 Sprouty‐4 inhibits transformed cell growth, migration and invasion, and epithelial‐mesenchymal transition, and is regulated by Wnt7A through PPARgamma in non‐small cell lung cancer. Mol. Cancer Res. 8:833–843. 20501643 10.1158/1541-7786.MCR-09-0400 PMC2888899

[23] Tetsu, O. , and F. McCormick . 1999 Beta‐catenin regulates expression of cyclin D1 in colon carcinoma cells. Nature 398:422–426. 10201372 10.1038/18884

[24] Chen, B. , R. Zhao , C. H. Su , M. Linan , C. Tseng , L. Phan , et al. 2012 CDK inhibitor p57 (Kip2) is negatively regulated by COP9 signalosome subunit 6. Cell Cycle 11:4633–4641. 23187808 10.4161/cc.22887 PMC3562308

[25] Marine, J. C. 2012 Spotlight on the role of COP1 in tumorigenesis. Nat. Rev. Cancer 12:455–464. 22673153 10.1038/nrc3271

[26] Su, H. , W. Huang , and X. Wang . 2009 The COP9 signalosome negatively regulates proteasome proteolytic function and is essential to transcription. Int. J. Biochem. Cell Biol. 41:615–624. 18706515 10.1016/j.biocel.2008.07.008 PMC2628451

[27] Tao, Z. H. , J. L. Wan , L. Y. Zeng , L. Xie , H. C. Sun , L. X. Qin , et al. 2013 miR‐612 suppresses the invasive‐metastatic cascade in hepatocellular carcinoma. J. Exp. Med. 210:789–803. 23478189 10.1084/jem.20120153 PMC3620363

[28] Stassi, G. , M. Todaro , M. Zerilli , L. Ricci‐Vitiani , D. Di Liberto , M. Patti , et al. 2003 Thyroid cancer resistance to chemotherapeutic drugs via autocrine production of interleukin‐4 and interleukin‐10. Cancer Res. 63:6784–6790. 14583474

